# Antibacterial effects of microbial synthesized silver-copper nanoalloys on *Escherichia coli, Burkholderia cepacia, Listeria monocytogenes* and *Brucella abortus*

**Published:** 2018-06

**Authors:** Sheida Mohammadi, Nima Hosseini Jazani, Mehri Kouhkan, Leila Ashrafi Babaganjeh

**Affiliations:** 1 Students, Research Committee, Urmia University of Medical Sciences, Urmia, Iran; 2 Department of Microbiology, Faculty of Medicine, Urmia University of Medical Sciences, Urmia, Iran; 3 Faculty of Pharmacology, Urmia University of Medical Sciences, Urmia, Iran

**Keywords:** *Escherichia coli*, *Burkholderia cepacia*, *Listeria monocytogenes*, *Brucella abortus*, Ag-Cu nanoalloy

## Abstract

**Background and Objectives:**

Bacterial resistance is an emerging public health problem worldwide. Metallic nanoparticles and nanoalloys open a promising field due to their excellent antimicrobial effects. The aim of the present study was to investigate the antibacterial effects of Ag-Cu nanoalloys, which were biosynthesized by *Lactobacillus casei* ATCC 39392, on some of the important bacterial pathogens, including *Escherichia coli*, *Burkholderia cepacia, Listeria monocytogenes* and *Brucella abortus*.

**Materials and Methods:**

Ag-Cu nanoalloys were synthesized through the microbial reduction of AgNO_3_ and CuSO_4_ by *Lactobacillus casei* ATCC39392. Furthermore, they were characterized by Fourier-Transform Infrared Spectrometer (FTIR) and Field Emission Scanning Electron Microscopy (FESEM) analysis in order to investigate their chemical composition and morphological features, respectively. The minimum inhibitory and minimum bactericidal concentrations of Ag-Cu nanoalloys were determined against each strain. The bactericidal test was conducted on the surface of MHA supplemented with 1, 0.1, and 0.01 μg/μL of the synthesized nanoalloy. The antimicrobial effects of synthesized nanoalloy were compared with ciprofloxacin, ampicillin and ceftazidime as positive controls.

**Results:**

Presence of different chemical functional groups, including N-H, C-H, C-N and C-O on the surface of Ag-Cu nanoalloys was recorded by FTIR. FESEM micrographs revealed uniformly distributed nanoparticles with spherical shape and size ranging from 50 to 100 nm. The synthesized Ag-Cu nanoalloys showed antibacterial activity against *L. monocytogenes* PTCC 1298, *E. coli* ATCC 25922 and *B. abortus* vaccine strain. However, no antibacterial effects were observed against *B. cepacia* ATCC 25416.

**Conclusion:**

According to the findings of the present research, the microbially synthesized Ag-Cu nanoalloy demonstrated antibacterial effects on the majority of the bacteria studied even at 0.01 μg/μL. However, complementary investigations should be conducted into the safety of this nanoalloy for *in vivo* or systemic use.

## INTRODUCTION

Nanotechnology is a modern research field dealing with synthesis, manipulation, and use of particles the size of which ranges from 1 to 100 nm. Metal nanoparticles (NPs) are extensively employed in many different areas such as health care, cosmetics, environmental health, and medicine (1). Silver NPs (AgNPs) are increasingly utilized in medical fields due to their peculiar properties, including antibacterial, antifungal, antiviral, anti-inflammatory, anti-cancer, and anti-angiogenic agents (2). They appear to be alternative antibacterial agents because of their large surface-to-volume ratio and crystal structure. Moreover, they have the ability to overcome the problem of bacterial resistance to a wide range of antimicrobial agents such as antibiotics (3). On the other hand, it has been reported that copper and copper oxide NPs have high potential to kill bacterial cells and heal wounds (4–7).

A variety of techniques have been applied to synthesize NPs. These techniques are classified in three main groups, including chemical, physical, and bio-based methods (1). Several studies have indicated that physical and chemical methods for the synthesis of NPs use high radiations or concentrated stabilizing and reducing agents, being harmful to human and the environment. Additionally, these methods are expensive and eco-unfriendly (8–10). Accordingly, genuine attempts have been made to develop economic as well as eco-friendly methods to synthesize NPs by using living cells, including micro-organisms which are not harmful to the environment (11–16). Bacteria are considered a new green factory for the bio-synthesis of metallic NPs because they have a fast growth rate and extraordinary abilities to adapt to very different environmental conditions. Furthermore, they are inexpensive and abundant in the environment and their cultivation conditions can be easily controlled (17).

Bimetallic nanoalloys are clusters with two or more metallic elements (18). They have attracted many researchers’ interest because of their specific chemical and physical properties. Bimetallic NPs, such as Platinum-Ruthenium, Copper- Palladium, Platinum - Molybdenum and Gold -Silver are some examples of nanoalloys. Many show catalytic properties. The properties and application of metallic NPs mainly depend on their size and shape. Various shapes are reported for metal NPs including bar shaped, spherical, rods, cubes, and wires, and also in the form of nanocrystals with an unpredictable symmetry (19). Like many other NPs and nanoalloys, several important properties are attributed to Ag-Cu nanoalloys. Ag-Cu nanoalloys have previously been synthesized from their metal salts by chemical co-reduction. In addition, they have shown antibacterial activity against *Escherichia coli* and they also showed better antimicrobial activity in comparison with Ag – only or Cu- only NPs (20). Recently the green synthesis of Ag-Cu nanoalloys using a plant extract was performed by two synthesis methods including core–shell and Janus morphologies by reversing the order of precursors. However, they did not report any antibacterial effects (21).

*Escherichia coli* is one of the most important causes of several types of multi - drug resistant infections. The emergence of antimicrobial resistance has threatened the therapeutic treatment of *E. coli* infections in recent decades. On the other hand, in the present study, *E. coli* was chosen as a prototype of Gram-negative bacteria (22). *Listeria monocytogenes* is Gram-positive, food-borne pathogen which causes fatal infections in immunocompromised patients. Several studies reported isolation of resistant strains of *Listeria* from different sources to several antibiotics. Also, in several studies, *Listeria* was used as the prototype of gram positive bacteria (23–28). *Brucella abortus* is one of the causative agents of human brucellosis. In recent years, a number of studies have reported reduced susceptibility of *Brucella* species to antibiotics or even strains with multi-drug resistance (29–32). *Burkholderia cepacia* complex is capable of causing several types of opportunistic infections in hospitalized patients. This microorganism shows high intrinsic resistance and is found to be one of the most resistant bacteria in the clinical settings; therefore, these infections are very difficult to treat and, in many cases, result in death (33–35).

The antibacterial effects of synthesized Ag-Cu nanoalloys were evaluated on several Gram-positive and Gram-negative bacterial strains including *Staphylococcus aureus, Bacillus subtilis, Klebsiella pneumoniae, Pseudomonas aeruginosa* (ATCC27853), and *Acinetobacter calcoaceticus* (ATCC 23055) in our previous study (36). Hence, the aim of the present study was to investigate the antibacterial effects of Ag-Cu nanoalloys, which were biosynthesized by *Lactobacillus casei* (ATCC 39392), on a number of other important bacterial pathogens, including *E. coli, B. cepacia, L. monocytogenes* and *B. abortus.*

## MATERIALS AND METHODS

### Microorganisms.

The reference species of the following bacteria were obtained from the American Type Culture Collection (ATCC) and the Persian Type Culture Collection (PTCC) at the Iranian Research Organization for Science and Technology (IROST). The bacteria included *L. casei* ATCC 39392, *E. coli* ATCC25922, *B. cepacia* ATCC 25416, *L. monocytogenes* PTCC 1298, and *B. abortus* vaccine strain. *L. casei* ATCC 39392 was employed for the biosynthesis of Ag-Cu nanoalloys, and the other bacterial strains were utilized to test the antibacterial effects of NPs which were produced.

### Microbial synthesis of Ag-Cu nanoalloys.

Ag-Cu nanoalloys were synthesized via the reduction of AgNO_3_ and CuSO_4_ salts (both from Sigma Aldrich, USA). In brief, in order to produce Ag-Cu nanoalloys, *L. casei* ATCC39392 was cultivated for 24 h at 30°C in MRS broth (Liofilchem, Italy) which was supplemented with Tween-80. The aqueous mixture of copper and silver salts (with the final concentration of 75% Ag and 25% Cu) was added to the flask. After the incubation period with metal salts (48 h), formation of NPs at the bottom of the flask was investigated by changing the color of the MRS broth from yellow to dark brown. Centrifugation at 3,500 rpm for 5 minutes was applied to extract the nanoparticles, and then the product was filtered and washed three times with deionized water and dried at 40°C for 4 h.

### Characterization of Ag-Cu nanoalloys.

The microbially synthesized Ag-Cu nanoalloys were characterized by various analytical methods, including Fourier-Transform Infrared Spectrometer (FTIR) (Thermo Nicolet Nexus 670 Model) in order to investigate the chemical composition of the synthesized nanoalloys. The spectra were scanned in the range of 500 to 4,000 cm^−1^. Field Emission Scanning Electron Microscopy (FESEM) analysis was carried out to study morphological features such as the shape and size of the nanoalloys.

### Antimicrobial effects: Determination of Minimum Inhibitory Concentration (MIC) and Minimum Bactericidal Concentration (MBC) of Ag-Cu nanoalloys.

MICs and MBCs of Ag-Cu nanoalloys were determined for *B. cepacia* (ATCC 25416), *L. monocytogenes* (PTCC 1298), *E. coli* ATCC25922 and *B. abortus* vaccine strain. CLSI 2016 guideline for Macro dilution method was applied to evaluate the antimicrobial activity of Ag-Cu nanoalloys. Muller Hinton Broth (MHB) was utilized for the overnight cultivation of bacterial strains at 37°C. Subsequently, the bacteria were harvested after centrifugation, washed, and re-suspended in normal saline. The antibacterial effects of Ag-Cu nanoalloys were investigated in the range of 2–1.95 × 10^−3^ μg/μL by preparing two-fold dilutions in the test tubes. 1.5 × 10^6^ Colony Forming Units (C.F.Us) of each isolate was added to each test tube, and the turbidity of broth media was investigated after 24 h at 37°C. MIC and MBC were considered as the lowest concentration of the Ag-Cu nanoalloys, causing no turbidity in MHB or inhibiting the growth of more than 99.9% of the initial inoculum of bacteria in MHA, respectively. MHB medium without nanoalloy and un-inoculated MHB medium were considered as positive and negative controls, respectively (37, 38). All experiments were conducted in triplicate.

### Determination of MIC and MBC of ampicillin, ciprofloxacin and ceftazidime.

MICs and MBCs of ampicillin, ciprofloxacin and ceftazidime (sigma) were determined by the microdilution broth method for studied bacteria according to the CSLI 2016 guideline. Microbial suspensions were prepared and the concentration was adjusted to 0.5 McFarland turbidity standard. Two-fold serial dilutions of an initial antibiotic solution in the range of 0.512–0.0005 μg / μL were prepared in MHB. MICs and MBCs were determined as described for biosynthesized nanoalloys.

### Bactericidal test.

Ag-Cu nanoalloy was tested in order to investigate its bactericidal effect. The bacterial strains were cultivated on the surface of MHA supplemented with 1, 0.1, and 0.01 μg/μL of the synthesized nanoalloy. A MHA medium without nanoalloy was utilized as the negative control. Incubation was performed at 37°C for 24 h, and the number of the colonies was determined afterwards (39, 40). AgNO_3_ and CuSO_4_ were inserted in separate MHA plates, with the concentration of 1 μg/μL, which were used to compare these metal salts’ bactericidal effects with Ag-Cu nanoalloy. All experiments were repeated three times, and the mean values were considered as the results.

## RESULTS

### Characterization of Ag-Cu nanoalloy.

#### Color change in MRS culture medium.

Synthesis of the Ag-Cu nanoalloy in broth medium was monitored by investigating color change after adding metal salts with defined concentrations to the MRS broth containing *L. casei* ATCC39392 for 24 h at 30°C. Forty-eight hours after adding AgNO_3_ and CuSO_4_ to the broth, it’s color turned from yellow to dark brown, indicating the formation of Ag-Cu nanoalloys. However, no color change was observed in the absence of the bacterial culture.

#### Fourier-transform infrared spectroscopy.

**Fig. 1.**
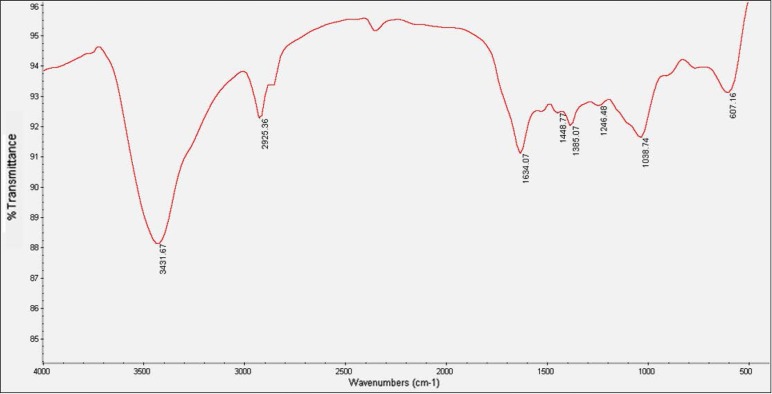
FTIR spectra of synthesized Ag-Cu nanoalloys

Presence of different chemical functional groups, including N-H stretching vibrations of amines or amide linkages (strong peaks at 3100–3680 cm^−1^), C-H stretching vibration of the alkenes group (small peak at 2925 cm^−1^), amide bands of proteins (at 1634 cm^−1^ and 1448 cm^−1^), C-N stretching vibrations of aromatic and aliphatic amines (the bands observed at 1335 and 1264 cm^−1^, espectively), and C-O stretching vibrations related to carboxylate and alcoholic groups (the weaker band at 1039 cm^−1^) on the surface of Ag-Cu nanoalloys were recorded by FTIR. Thus it can be concluded that the biosynthesized nanoalloys are coated by proteins. In addition, the coating of nanoalloys by biomolecules stabilizes them in water and prevents them from aggregation (Fig. 1).

#### Field emission scanning electron microscopy.

**Fig. 2.**
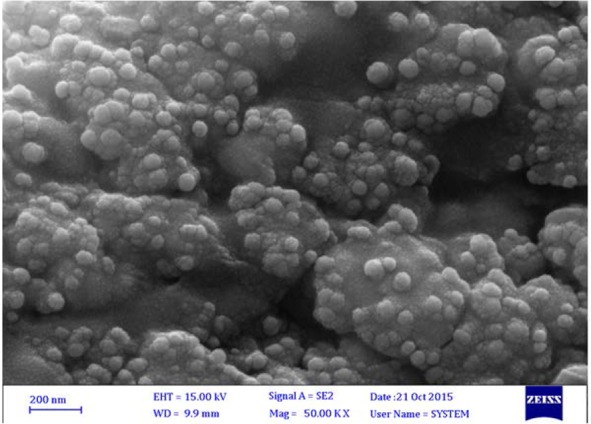
FESEM image of synthesized Ag-Cu nanoalloys

Fig. 2. shows FESEM micrographs of the biosynthesized nanoalloy and analyzes the size, shape, and distribution of the nanoparticles. It can be concluded from this figure that the size of the synthesized nanoalloys are less than 100 nm, that these nanoalloys have spherical shape, and that they are uniformly distributed.

#### Minimum inhibitory and minimum bactericidal concentrations of Ag-Cu nanoalloys.

The synthesized Ag-Cu nanoalloys showed antibacterial activity against *L. monocytogenes* PTCC 1298, *E. coli* ATCC 25922, and *B. abortus* vaccine strain. However, no antibacterial effects were observed against *B. cepacia* ATCC 25416. Table 1 summarizes the results of MIC and MBC determination for the bacteria tested and compares these effects with some selected antibiotics.

**Table 1.** MIC and MBC of Ag-Cu nanoalloys for microorganisms tested in comparison with selected antibiotics.

**Strain**	**MIC, (MBC) Ag-Cu nanoalloys μg /μL Mean ± SD**	**MIC, (MBC) ciprofloxacin μg /μL Mean ± SD**	**MIC, (MBC) Ampicillin μg /μL Mean ± SD**	**MIC, (MBC) Ceftazidime μg /μL Mean ± SD**
*L. monocytogenes* PTCC 1298	0.250±0, (0.250±0)	.004(0.004)	0.004 ± 0.0 (0.004 ± 0.0)	Nt
*B. abortus* vaccine strain	0.250±0, (0.250±0)	0.008 ± 0.0 (0.008 ± 0.0)	Nt	Nt
*B. cepacia* ATCC 25416	− (−)	Nt	Nt	.032 ± 0.0 (0.32 ± 0.0)
*E. coli* ATCC25922	0.500±0, (0.250±0)	0.004 ± 0.0 (0.004 ± 0.0)	0.008 ± 0.0 (0.008 ± 0.0)	Nt

Nt: Not tested

#### Bactericidal test.

The antibacterial tests were performed on the selected bacterial strains on MHA agar plates containing different concentrations of Ag-Cu nanoalloys. As shown in Table 2, 0.5, 0.1, and 0.01 μg/μL concentrations of the synthesized nanoalloys completely prevented the growth of *L. monocytogenes*. For the *B. abortus* vaccine strain and *E. coli*, 0.5 and 0.1 μg/μL concentrations of Ag-Cu nanoalloys were completely inhibitory; however, fewer than ten colonies were observed in the presence of 0.01 μg/μL for both of the abovementioned strains. No antibacterial effects were observed on *B. cepacia* in the presence of all of the concentrations tested (Table 2). AgNO_3_ and CuSO_4_, with equal concentrations of the nanoalloys, had no inhibitory effects on all of the bacterial strains used as controls.

**Table 2.** Number of bacterial colonies grown on MHA plates containing 0.5, 0.1 and 0.01 μg/μL of Ag-Cu nanoalloys

**Bacterial strains**	**Inoculum size C.F.U**	**No. of colonies on positive control plate**	**Nanoalloy concentration (NC) μg /μL**		
			**0.5**	**0.1**	**0.01**
*L. monocytogenes* PTCC 1298	10^4^	++++	NG	NG	NG
*B. abortus* vaccine strain	10^4^	++++	NG	NG	+
*B. cepacia* ATCC 25416	10^4^	++++	++++	++++	++++
*E. coli* ATCC25922	10^4^	++++	NG	NG	+

+<10 C.F.U

++<100 C.F.U

+++<1000 C.F.U

++++>1000 C.F.U

** Treatment: (AgNo_3_ 75%, CuSo_4_ 25%)

## DISCUSSION

Antimicrobial agents introduced thus far are synthetic, semisynthetic, or completely natural. Antibiotics, as a group of antimicrobials, are miracle drugs with selective toxicity which are usable in the human body via systemic or topical prescription. However, the misuse of antibiotics has resulted in development of multi-drug-resistant microorganisms, one of the most threatening challenges to humans, especially in health care settings. Therefore, the use of new technologies appears to be essential for designing unique effective antibiotics. Nanotechnology is a promising field which can overcome the problem of antibiotic resistance. Several metallic nanoparticles have been developed to eradicate or inhibit antibiotic-resistant bacteria. Furthermore, the microbial synthesis of nanoparticles and nanoalloys is a friendly process in both fields of environment and economy and does not use toxic chemical compounds in the synthesis procedure; however, the safety of *in vivo* use of these components should be considered (41). The high antibacterial effects and low toxicity of silver and copper nanoparticles have been previously demonstrated (4, 42–44).

Taner et al. (2011) reported the synthesis of Ag-Cu nanoalloys by chemical co-reduction of their metal salts in an aqueous solution with hydrazine hydrate and investigated their antibacterial effects on *E. coli*. They found that Ag-Cu nanoalloys have better antibacterial effects in comparison with silver or copper nanoparticles alone (20). In the present study, Ag-Cu nanoalloys were synthesized biologically, using a *Lactobacillus* strain, and their antibacterial effects on *E. coli* as well as some other clinically important bacterial strains were evaluated. Our results also revealed notable antibacterial effects of Ag-Cu nanoalloys on *L. monocytogenes, E. coli* and *B. abortus*.

Many antibacterial agents should penetrate into the bacterial cells as they interfere with intracellular functions. In Gram-negative bacteria, the outer membrane provides an additional barrier that must be traversed by antimicrobials (45). Gram-negative bacteria have shown significant variation in permeability to antibiotics; however, unlike *E. coli,* a number of Gram-negative bacterial species such as *Burkholderia* are considered “impermeable” (46). The composition of the outer membrane of Gram-negative bacteria has strong effects on its permeability to antimicrobial agents. It has been reported in several studies that the *B. cepacia* complex is intrinsically resistant to different classes of antibiotics. Numerous mechanisms are involved in the resistance of *Burkholderia* to antimicrobials; for example, the outer membrane in *Burkholderia* species contains a trimeric porin called Omp38. Omp38 is homologue to OmpF of *E. coli*, but less permeable than OmpF. This, along with other mechanisms such as the presence of multiple efflux pumps (46), may result in the resistance of *Burkholderia* species to many antibiotics and antimicrobial agents.

It has been indicated that AgNPs cause severe damage to the membrane and result in cytosolic leakage in *B. pseudomallei* which is the causative agent of melioidosis (47). In another study, carbon NPs complexed with Ag were found to have effective antimicrobial activity against some multi-drug-resistant bacteria, including *B. cepacia* (48). Nevertheless, to the best of our knowledge, the antibacterial effects of nanoalloys on *B. cepacia* have not been investigated yet. We found that Ag-Cu nanoalloys did not have any antimicrobial effects on *B. cepacia* (ATCC 25416). Several studies have mentioned that infections resulting from *B. cepacia* in individuals with underlying conditions, especially in cystic fibrosis patients, remain challenging in respect to their treatment (49). As a result, investigations into newer antibacterial agents for *B. cepacia* complex infections are recommended.

The MICs and MBCs of ciprofloxacin for *L. monocytogenes, B. abortus* and *E. coli*, ampicillin for *L. monocytogenes* and *E. coli* and ceftazidime for *B. cepacia* were compared with Ag-Cu nanoalloy in the present study. As the results of the present study showed, selected antibiotics were more effective than synthesized nanoalloy against tested microorganisms. Unlike antibiotics (50), developing resistance to NPs requires multiple simultaneous gene mutations in the same bacterium due to involvement of several simultaneous mechanisms of action of NPs. So, NPs should be considered as valuable alternatives to antibiotics which have high potential to solve the problem of the emergence of multi-drug resistant microorganisms (51). On the other hand, several characteristics including the size, charge, surface morphology, and crystal structure are significant factors that regulate the antibacterial effects of NPs (51). Improvement of synthesis methods may increase the antibacterial efficacy of the produced Ag-Cu nanoalloy by altering the above mentioned features.

In conclusion, the findings of the present research confirmed that microbially synthesized Ag-Cu nanoalloy showed notable antibacterial effects on a number of bacteria studied. However, complementary investigations should be conducted into the safety of this microbially synthesized Ag-Cu nanoalloy for *in vivo* or systemic usage.
